# Characteristics, Therapeutic Approaches and Outcomes of Patients Older than 80 Years Old with Metastatic Colorectal Cancer Compared with Younger Patients

**DOI:** 10.3390/jcm14041099

**Published:** 2025-02-09

**Authors:** Melissa R. Yeo, Ioannis A. Voutsadakis

**Affiliations:** 1 Northern Ontario School of Medicine, Sudbury, ON P3E 2C6, Canada; meyeo@nosm.ca; 2 Algoma District Cancer Program, Sault Area Hospital, Sault Ste. Marie, ON P6B 0A8, Canada; 3 Section of Internal Medicine, Division of Clinical Sciences, Northern Ontario School of Medicine, Sudbury, ON P3E 2C6, Canada

**Keywords:** colorectal cancer, metastatic, octogenarians, chemotherapy, surgery, radiation therapy, metastasis

## Abstract

**Background:** While advances in therapies have improved metastatic cancer survival rates, elderly patients with colorectal cancer often experience delayed diagnoses, receive less frequent systemic therapies, and show inferior survival outcomes compared to younger groups. Patients over the age of 80 years old face greater treatment risks due to frailty and comorbidities. In this article, we examine characteristics, treatment and outcomes in older adults with metastatic colorectal cancer. **Methods:** The medical records of all patients aged 80 years and above and comparable patients aged 65–75 years old, who were diagnosed with stage 4 colorectal cancer at a cancer center over a six-year period, were retrospectively reviewed. **Results:** Patients in the 80 years old and older group more frequently had right-sided primary colon cancer (71.5%), compared to younger patients aged 65–75 years old (34.1%, *p* = 0.006). Patients in the younger cohort more commonly presented with stage 4 disease at initial diagnosis (59.5%) compared to older patients (22.2%). Elevated carcinoembryonic antigen (CEA) levels were more commonly identified in younger metastatic patients (76.3% vs. 46.4%, *p* = 0.013). Patients in the younger age group were more likely to have received previous neoadjuvant and adjuvant chemotherapy prior to metastatic progression (*p* = 0.02, and *p* = 0.01); however, a significant difference in palliative chemotherapy was not identified between the age groups of metastatic patients. The adverse effects of chemotherapy treatment were similar between the age groups. **Conclusions:** The active treatment of metastatic colorectal cancer in patients aged 80 and above is feasible when tailored according to the patients’ performance status, comorbidities, and life expectancy. Understanding metastatic disease presentations in elderly patients can improve treatment outcomes in this challenging-to-treat group.

## 1. Introduction

Colorectal cancer is a prevalent gastrointestinal malignancy, and its prevalence and associated morbidity and mortality have remained high despite efforts for early diagnosis and treatment, with 2 million patients diagnosed with the disease annually worldwide [1]. Colorectal cancer is currently the third most common cancer in terms of incidence after lung and breast cancer and the second most lethal cancer globally after lung cancer. Recent decades have witnessed progress in colorectal cancer treatments that have led to improved survival outcomes for patients with both localized and metastatic disease [2]. New targeted therapies and immunotherapies have entered the therapeutic armamentarium for a subset of colorectal cancer patients, but many patients remain with only cytotoxic chemotherapies as a systemic treatment option, and this is true in both the early and metastatic settings. As is common with many cancer types, colorectal cancer is a disease of the older population, with the incidence increasing with increasing age [2]. The incidence of colorectal cancer is 66.1 per 100,000 people in those between 55 and 60 years old, and this increases to 214.7 per 100,000 people for those 80 to 85 years old and to 234.7 per 100,000 people for those above the age of 85 years old [2]. Older patients suffer from a higher prevalence of comorbid conditions and from frailty, which complicate their ability to receive cancer treatments for their colorectal cancer, as these therapies tend to be more toxic [3]. Given this prevalence of frailty and comorbidities, many treating physicians consider older age, over 80 years old, a strong contraindicating factor for extensive operations and cytotoxic chemotherapies [3].

Oligometastatic colorectal cancer with localization to the liver or lung has been treated with metastasectomies in these two organs, which have become increasingly feasible with improvements in surgical techniques and perioperative supportive care [4]. However, these surgeries remain extensive and, for patients diagnosed with de novo metastatic disease, need to be associated with resection of the primary tumor in order to meaningfully improve survival outcomes. Therefore, they are feasible only for a minority of older patients [4]. Systemic treatment for metastatic colorectal cancer relies on cytotoxic chemotherapy with the addition of monoclonal antibodies targeting angiogenesis or the tyrosine kinase receptor EGFR for patients with a wild-type KRAS oncogene [5]. A minority of patients may have additional options available based on the genomic characteristics of their disease. These include immune checkpoint inhibitors for patients with mismatch repair-deficient/high-microsatellite-instability disease, combinations of BRAF inhibitors with anti-EGFR antibodies in patients with BRAF V600E-mutated cancers, HER2-targeting therapies in patients with colorectal cancers overexpressing HER2, and NTRK inhibitors in more rare cases with NTRK alterations [6,7,8]. Targeted therapies may be less toxic and better tolerated than cytotoxic chemotherapy for elderly patients, although they are not devoid of their own toxicities [9,10]. Moreover, only a minority of patients possess the targeted alterations that would make them candidates for these potentially less toxic therapies.

In the current research, we investigated the characteristics, therapeutic modalities and survival outcomes of patients 80 years old and older diagnosed with metastatic colorectal cancer in a single cancer center, and we compared these patients with younger patients aged 65 to 75 years old diagnosed with metastatic colorectal cancer. Although this age group represents a non-negligible percentage of colorectal cancer patients in most oncology practices, its characteristics and specific therapeutic needs are not extensively described in the literature. The current study aims to cover this gap by improving our understanding of the differences that characterize the disease in younger and older patients and identifying potential factors to optimize outcomes.

## 2. Patients and Methods

This investigation consisted of a retrospective review of patient case records with the inclusion of patients over 80 years old treated for colorectal cancer in a single cancer center between 2017 and 2023. Electronic records were evaluated, and information on the demographic and tumor characteristics of the included patients was extracted and analyzed. A group of patients with metastatic colorectal cancer aged 65 to 75 years old treated in the same period were matched by date of diagnosis and included as comparison controls. Patient demographic data were extracted from medical records, as well as information on the location, grade and stage of tumors, laboratory results including tumor markers, such as carcinoembryonic antigen (CEA), initial patient presentation and comorbidities. Treatments and outcome measures, including surgical treatments, neoadjuvant and adjuvant chemotherapy for previous localized disease, the dose of chemotherapy administered and resulting adverse effects, and radiation therapy were also extracted from electronic medical records. Overall survival was calculated as the time from the diagnosis of metastatic colorectal cancer, as confirmed by a positive biopsy or radiologic study, to the death of the patient, or alternatively changed to the time of the last follow-up. Progression-free survival was calculated as the time from diagnosis to the time of confirmed radiologic or clinical disease progression or, if there was no documented progression, to the time of the last follow-up without progression.

Descriptive statistics were computed from the database’s primary data, and the calculation of measures of central tendency and spread was presented to summarize variables of clinical interest. The evaluation of statistical differences in categorical clinical and laboratory parameters was performed with the χ^2^ test or Fisher’s exact test, and the t test was used to evaluate differences in continuous variables of interest. The Kaplan–Meier method was applied for the construction of overall survival and progression-free survival curves, and the Log Rank test was employed to compare the survival curves of metastatic colorectal cancer patients over 80 years old and their counterparts aged 65 to 75 years old. All *p* values derived from the statistical comparisons were considered significant if *p* < 0.05. Missing data were rare in most parameters of interest, and only a few parameters, such as tumor grade and glucose values, were missing in a large number of patients. As such, no sensitivity analysis for missing values was performed. The study protocol obtained regulatory approval by the Research Ethics Board of the institution (#2021-05-01).

## 3. Results

Thirty-two patients aged 80 years old and older (older age group) were diagnosed with stage 4 colorectal cancer and were included in the analysis. A similar group of 41 metastatic colorectal cancer patients, aged 65–75 years old (younger group), were identified over the same period according to the date of diagnosis. The mean age of the older group was 83 years old (range: 80–91 years old). The mean age of the younger group was 70 years old (range: 65–74 years old) (Table 1). There were no significant differences between the groups in terms of family history of colon cancers or of other types of cancer. There were some differences in the symptoms at the initial presentation of the disease, which, however, were not statistically significantly different between the two age groups, with bleeding/anemia being the most common presentation observed in those 80 years old and above (53.1%), followed by screening (31.3%) and bowel changes/obstruction (15.6%). Younger patients were most commonly diagnosed through screening measures (35.9%), followed by bleeding/anemia (33.3%) and bowel changes/obstruction (30.8%). A significantly higher percentage of patients in the older group presented with right-sided colon cancers (71.5%), compared to the younger cohort (34.1%, *p* = 0.006). Rectosigmoid and rectal tumors were the next most common in the older group (21.4%), with left-sided cancers less frequently diagnosed in both age groups (older group: 7.1%; younger group: 31.7%). Patients who presented at initial diagnosis with stage 4 tumors were significantly more common in the younger age group (older group: 22.2%; younger group: 59.5%, *p* = 0.03), whereas patients in the older group were more commonly diagnosed with stage 3 disease, which later progressed to metastatic disease (older group: 55.6%; younger group: 27.0%, *p* = 0.036). The older group most commonly presented with grade 1 disease (41.6%), whereas the younger group commonly presented with grade 2 tumors (47.1%), although the overall differences in grade did not reach statistical significance (Table 1).

In comparing laboratory values at the time of diagnosis with metastatic colorectal cancer, patients in the 65–75 age group had a higher percentage of elevated CEA (CEA above 4.7 μg/L in 76.3% of patients in the group) compared to metastatic patients aged 80 years old and above (CEA above 4.7 μg/L in 46.4%, *p* = 0.013). Elevated creatinine levels were significantly more common in the older age group compared to the younger cohort (older group: 20%; younger group: 2.6%, *p* = 0.02), suggesting decreased renal function reserves in the older group. There were no significant differences in the levels of lactate dehydrogenase (LDH), serum albumin, platelets, or glucose levels between the two age groups (Table 2). Overall, the baseline clinical and laboratory characteristics that significantly differed between the two groups included the sidedness of the primary disease, the stage at initial presentation, and CEA and creatinine levels.

The treatments received in the adjuvant setting for patients who were initially diagnosed with localized colorectal cancer were analyzed to decipher differences between the age groups (Table 3). Significantly more patients in the younger age group received neoadjuvant chemotherapy for rectosigmoid and rectal tumors compared to the older patient group (older group: 16.7%; younger group: 71.4%, *p* = 0.02). Patients with an initial diagnosis of stage 2 cancer were more likely to have received adjuvant chemotherapy if they were in the younger cohort (older group: 20%; younger group: 100%, *p* = 0.01). However, no difference in the use of adjuvant chemotherapy was identified in patients with a previous stage 3 disease. For patients who presented with stage 4 disease or progressed to metastatic disease after initial diagnosis and treatment, no statistically significant differences were identified in the rates of palliative chemotherapy treatments, although a trend for more frequent chemotherapy administration in the younger group was evident (older group: 34.4%; younger group: 53.7%, *p* = 0.10). The number of lines of palliative chemotherapy delivered also showed a nonsignificant trend in the younger age group (*p* = 0.10). For radiation therapy, there was not a significant difference between the groups for both previous neoadjuvant/adjuvant therapy and palliative radiation (*p* = 0.36, and *p* = 0.57, respectively). Patients in the older age group were more likely to have received a previous curative surgery for localized cancer that then progressed to metastatic disease compared to metastatic patients in the younger age group (older group: 78.1%; younger group: 47.6%, *p* = 0.01) (Table 3).

In metastatic patients who received palliative chemotherapy, there was no significant difference in the adverse effects of chemotherapy treatments between the older and younger age groups (Table 4). Fatigue, neutropenia, peripheral neuropathy, hand and foot syndrome or other skin toxicities, bowel changes (including diarrhea, and constipation), nausea/vomiting, mucositis, and other events (fatigue, shortness of breath, bleeding) were reported at similar rates in both patients aged over 80 years old and those aged 65–75 years old who received chemotherapy treatments (Table 4).

In comparing the comorbidity burden between the older and younger age groups, obesity (BMI > 30) was significantly more prevalent in patients aged 65–75 years old (older group: 12.5%; younger group: 43.9%, *p* = 0.005). Cardiovascular and neurovascular disease was significantly more common in the older age group (older group: 65.6%; younger group: 26.8%, *p* = 0.002), as was dyslipidemia (older group: 75.0%; younger group: 39.0%, *p* = 0.004). There was no significant difference in the prevalence of diabetes or hypertension between the two age groups (Table 5).

**Figure 1:**
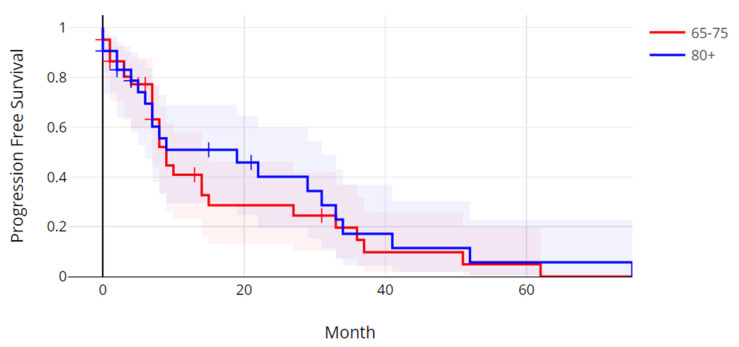
Progression-free survival of patients with metastatic colorectal cancer over 80 years old versus patients 65 to 75 years old. Log Rank *p* = 0.59.

**Figure 2:**
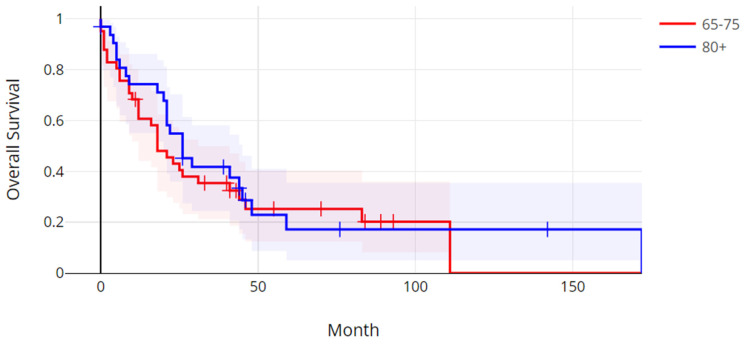
Overall survival of patients with metastatic colorectal cancer over 80 years old versus patients 65 to 75 years old. Log Rank *p* = 0.52.

The median progression-free survival in the older age group was 5.58 months (range: 0.3–75.7 months), and the median progression-free survival in the younger age group was 7.47 months (0.3–62.9 months). There was not a statistically significant difference in progression-free survival between the two age groups (Log Rank test *p* = 0.59) (Figure 1). The median overall survival (OS) in the older group was 26.3 months (range: 0.7–172.5 months), and the median OS in the younger age group was 18.8 months (range: 0.4–111.4 months). The overall survival between the two age groups was also not significantly different (Log Rank test *p* = 0.52) (Figure 2).

## 4. Discussion

The increasing age of populations in Western countries has been a constant trend in recent decades, increasing the prevalence of most common cancers, which are more prevalent in older patients. The average age of diagnosis for many of these cancers is around or greater than 65 years old, and about one in six patients are diagnosed with cancer at an age older than 80 years old [11]. Patients older than 80 years old often do not receive the standard of care therapies for their specific type and stage of cancer due to the unwillingness of providers to expose them to toxic treatments or a perception among patients and families of the futility of these treatments in the older group. This was shown in a Canadian study, where significantly fewer patients aged 80 or older had a surgical, medical oncology and radiation oncology consultation for their cancer compared to cancer patients aged 65 years old and younger [11]. Although these differences may stem from a number of reasons and, in some cases, consultations may even be inappropriate if patients are not clinically well enough to receive treatments, on most occasions, an informed decision would benefit from a formal consultation. In metastatic colorectal cancer, up until recently, treatment has relied mainly on systemic cytotoxic chemotherapy. Unfortunately, this treatment can be associated with severe adverse side effects, which tend to be more prevalent with combination regimens and with increasing age [12]. Moreover, with our improved understanding of the molecular biology of colorectal cancer, several targeted treatments have been added to the therapeutic armamentarium of systemic therapies and may be used to supplement or replace cytotoxic chemotherapy. These include immunotherapy with immune checkpoint inhibitors for high-microsatellite-instability cancers, combinations of anti-EGFR antibodies with BRAF inhibitors for patients with *BRAF* mutations, combinations of anti-EGFR antibodies with KRAS inhibitors for patients with C12S *KRAS* mutations, and targeted HER2 therapies for patients with colorectal cancers bearing *ERBB2* amplifications or overexpression [6,7,8,13]. Targeted therapies have their own adverse side effects but are, in many occasions, better tolerated and potentially more appropriate for patients who, due to age or comorbidities, would be poor candidates for cytotoxic chemotherapy [14]. Despite these new options, the majority of metastatic colorectal cancer patients do not possess targetable alterations, and therefore, cytotoxic chemotherapy remains the only effective therapeutic alternative. The prediction of high-grade toxicity in the elderly is an important aspect of the evaluation of geriatric patients considered for cancer therapy [15]. Various tools have been introduced for chemotherapy toxicity prediction in older patients, and these may help with difficult discussions regarding active cancer therapy initiation in the older population. The most widely cited tool was proposed by the Cancer and Aging Research Group (CARG) and has been endorsed by the American Society of Clinical Oncology (ASCO) guidelines [16,17]. According to this tool, geriatric cancer patients over the age of 70 years old are categorized into a low, intermediate or high risk for chemotherapy toxicity group [16]. Eleven demographic, tumor and functional factors are used in the calculation of the risk category. These include age above 72 years old, the type of primary cancer, with gastrointestinal or genitourinary cancers considered to be at higher risk of toxicity, the dose and number of chemotherapy drugs in the planned regimen, low hemoglobin, renal dysfunction, hearing loss, falls in the last 6 months, inability to take medications independently, mobility restrictions and social activity, with isolation being a risk factor [16]. Each parameter contributes 1 to 3 points, and the sum of the points obtained determines the risk for toxicity category assigned to the patient. Another prediction tool called Index4 is calculated based on only four parameters, including the Eastern Co-operative Oncology Group (ECOG) performance status of the patient, low serum albumin, renal insufficiency and the presence of a metastatic cancer [18]. This tool has the advantage of assigning patients to only two categories of risk, which may be more practical. Index4 was shown to perform similarly to the CARG tool in a study from a single center but has not been validated as extensively. In addition to the ASCO, other international oncology societies advocate for the use of formal clinical assessments in the older population. For example, The European Society of Medical Oncology (ESMO) and the International Society of Geriatric Oncology (SIOG) have endorsed the use of a geriatric assessment to optimize the management of geriatric cancer patients [19].

In the current study, elderly metastatic colorectal cancer patients over 80 years old did not differ significantly from younger metastatic colorectal cancer patients diagnosed in the same time period in terms of their sex, family history of cancer and the presenting symptoms. However, right colon primaries and initial presentation with localized disease were more prevalent in the older group. As a result, older patients had more frequently undergone a previous surgery for resection of the primary tumor. In contrast, patients in the older group had less frequently received previous adjuvant chemotherapy for stage 2 cancers and neoadjuvant chemotherapy for rectal cancers. A trend toward a lower use of palliative chemotherapy was also observed in the elderly. Older patients more frequently had cardiovascular comorbidities and reduced renal function. The prevalence of thrombocytosis, which was examined as a marker of inflammation, was not different between the groups. This may be explained by the fact that platelet counts are affected by a variety of inflammatory and bone marrow conditions beyond the presence of cancer [20,21]. Despite these differences, neither the OS nor the PFS differed significantly between the two metastatic colorectal cancer age groups.

In our series, most patients in the older group had been diagnosed with initial localized cancer and undergone curative surgery before relapsing with metastatic disease. Therefore, age had not prevented them from being treated with curative intent. Surgical resection, when appropriate, can be facilitated in this age group with minimally invasive procedures, which have become available with advances in surgical techniques [22]. The less frequent use of palliative chemotherapy in the elderly has been shown in other series and may contribute to worse outcomes [23]. Careful selection based on comorbidities and organ reserves, in addition to age, may help increase the percentage of elderly patients that could be candidates for these potentially beneficial therapies. Moreover, the introduction of targeted systemic therapies, which tend to be better tolerated, may also facilitate systemic therapy administration in the elderly with metastatic colorectal cancer.

Although early-stage colorectal cancers are cured with surgical resection with or without adjuvant chemotherapy or chemoradiotherapy, metastatic disease is incurable in most cases [24]. It can be palliated with systemic treatments, which, in all but a few cases, currently encompass cytotoxic chemotherapy. The most effective chemotherapy regimens used in the metastatic colorectal cancer setting involve combinations of a fluoropyrimidine with oxaliplatin or irinotecan and a monoclonal antibody targeting VEGF or, in patients with wild-type *KRAS*, targeting EGFR [5]. In patients over the age of 80 years old, these regimens could cause significant toxicity and can be administered only in carefully selected patients with preserved organ function reserves and no or few comorbidities, after discussion with the patient and family. Regimens that include only one cytotoxic agent, usually a fluoropyrimidine and a targeted monoclonal antibody, and are usually reserved for later palliative treatment are more appropriate for most octogenarians who wish to pursue active palliative colorectal cancer treatment. Additional options include oral receptor tyrosine kinase inhibitors, such as regorafenib and fruquitinib, which mainly target VEGFR, and targeted therapies that are appropriate for patients with specific genomic alterations, including microsatellite instability, *BRAF* V600 mutations, *KRAS* G12C mutations, *ERBB2* amplifications or overexpression, and NTRK receptor fusions [5]. Therefore, a genomic evaluation is mandatory for elderly colorectal cancer patients, as in younger patients. Targeted agents, although not devoid of their own adverse effects, are, in general, less toxic and better tolerated by the elderly. As, with our improved understanding of the molecular pathogenesis of colorectal cancer, options for targeted therapies will undoubtedly increase, it is important for older patients to be referred for oncologic consultations to optimize their care.

The location of the primary cancer in the proximal or distal colon is of biological and clinical importance, as cancers originating from different parts of the colon have distinct molecular alterations [25]. Proximal cancers more often have high microsatellite instability due to epigenetic alterations, most commonly hypermethylation of the promoter of the *MLH1* gene [26]. Similarly to our results, some studies have reported a higher prevalence of proximal colon primary cancers in older patients, although other studies have not confirmed this observation [12,27,28]. Therefore, elderly patients have at least an equal and possibly higher probability than younger patients of possessing the high-MSI phenotype and can benefit from immune checkpoint inhibitor treatment. A subset of patients receiving immune checkpoint inhibitors are long-term responders, further underlying the need to identify patients who are candidates for these therapies. Another molecular alteration that is associated with immune checkpoint inhibitor response is the hyper-mutated phenotype associated with mutations in the catalytic domain of proofreading polymerases epsilon (POLE) and delta (POLD1) [29]. Genomic series have observed no significant differences in the prevalence of common colorectal cancer-associated mutations, such as *APC*, *KRAS*, *PIK3CA* and *TP53*, in octogenarians compared with younger colorectal cancer patients [30,31,32]. Some studies have reported a higher prevalence of *BRAF* mutations in older patients [31]. These mutations are associated, in some cases, with high-MSI tumors.

Another pathogenic axis in colorectal cancer that may differ in older and younger colorectal cancer patients is the gut microbiome [33]. Colorectal cancer tissues differ from normal bowel tissues in terms of the composition of the gut microbiome, with species such as *Bacteroides fragilis* and *Escherichia coli* being more abundant in cancer and causing dysbiosis in the host and cancer-promoting inflammation [34]. Moreover, age was a factor in the diversification of the tumor microbiome in colorectal cancer patients, with younger patients having tumors with less common pathogenic species, such as other bacteroides species besides *B. fragilis*, while older patients had a decreased prevalence of protective species, such as *Lachnospira eligens* and *Streptococus salivarius* [33]. Fecal microbiota transplantation, a procedure providing protective species in patients with various conditions, could be of interest as a preventive and therapeutic strategy in colorectal cancer if further studies confirm its efficacy [34,35].

One of the main limitations of our study concerns the retrospective nature of the investigation, which relied on a review of electronic medical records and, as such, depended on the quality and completeness of the documentation, similarly to all studies employing a retrospective methodology. In addition, the cohorts reported were treated at a single cancer center, which may have introduced bias related to the approaches used in that center not necessarily pertaining to those used in other cancer clinics. On the other hand, the treatment and follow-up of the patients at a single center allowed for a more complete outcome evaluation. This study is one of the few studies reporting cohorts of metastatic colorectal cancer patients in the older old population. However, the number of patients examined, despite including all octogenarians and older patients with colorectal cancer treated in the time period of the study, was rather small. Genomic evaluation of microsatellite instability/mismatch repair status and other common colorectal cancer mutations had not been performed in the cohort and therefore is not included in the data presented. As mentioned above, genomic studies from the TCGA and other groups have observed a similar prevalence of mutations in tumor suppressor genes *APC* and *TP53* and in oncogenes *KRAS* and *PIK3CA* in older and younger colorectal cancer patients [30,31,32].

In conclusion, octogenarians and older patients with metastatic colorectal cancer present specific dilemmas for physicians involved in their care. Metastatic colorectal cancers in these patients have specific characteristics and pose particular therapeutic conundrums. Although the options available for well-tolerated systemic therapies have increased in recent decades and multidisciplinary approaches in active and supportive treatments have improved, older patients have comorbidities and reduced organ functional reserves, often precluding all but the least toxic therapies. Effective palliation of cumbersome symptoms and an approach for preserving quality of life, with patients’ wishes being central to treatment considerations, are essential in the care of the older population. Reassuringly, as reported in our cohort, a tailored treatment approach in the older group provides survival outcomes comparable to those in younger metastatic colorectal cancer patients. The different therapeutic options available to younger patients could potentially benefit older patients and could be discussed on an individual basis, with an open mindedness that considers the patient as a whole, beyond their numeric age. This approach would avoid stereotypes that could deprive elderly patients of beneficial treatments for improving quality and extending their quantity of life.

## Figures and Tables

**Table 1:** Patient and disease characteristics in patients with metastatic colorectal cancer over 80 years old versus patients 65–75 years old. NA: not available.

Characteristic	All (%) (*n* = 73)	Group > 80 Years Old (%) (*n* = 32)	Group 65–75 Years Old (%) (*n* = 41)	*p*
Mean Age (Range)		83 (80–91)	70 (65–74)	
Gender				
Male	41 (56.2%)	20 (62.5%)	21 (51.2%)	0.42
Female	32 (43.8%)	12 (37.5%)	20 (48.8%)	
Family History of Colon Cancer				
Yes	12 (16.4%)	5 (15.6%)	7 (17.1%)	0.87
No	61 (83.6%)	27 (84.4%)	34 (82.9%)	
Family History of Other Cancers				
Yes	39 (53.4%)	17 (53.1%)	22 (53.7%)	0.96
No	34 (46.6%)	15 (46.9%)	19 (46.3%)	
Presentation of Disease				
Incidental/Screening	24 (33.8%)	10 (31.3%)	14 (35.9%)	0.18
Anemia/Bleeding	30 (42.3%)	17 (53.1%)	13 (33.3%)	
Bowel Changes/Obstruction	17 (23.9%)	5 (15.6%)	12 (30.8%)	
N/A	2	0	2	
Location of Tumor				
Right	34 (49.3%)	20 (71.5%)	14 (34.1%)	0.006
Left	15 (21.7%)	2 (7.1%)	13 (31.7%)	
Rectal/ Rectosigmoid	20 (29.0%)	6 (21.4%)	14 (34.1%)	
NA	4	4	0	
Stage at Initial Diagnosis				
1	1 (1.5%)	1 (3.7%)	0	0.03
2	10 (15.6%)	5 (18.5%)	5 (13.5%)	
3	25 (39.1%)	15 (55.6%)	10 (27.0%)	
4	28 (43.8%)	6 (22.2%)	22 (59.5%)	
NA	9	5	4	
Grade of Tumor				
1	13 (31.7%)	10 (41.6%)	3 (17.6%)	0.25
2	15 (36.6%)	7 (29.2%)	8 (47.1%)	
3	13 (31.7%)	7 (29.2%)	6 (35.3%)	
NA	32	8	24	

**Table 2:** Laboratory evaluations at initial metastatic disease presentation in patients over 80 years old versus patients aged 65 to 75 years old. NA: not available.

	All (%) (*n*= 73)	Group > 80 Years Old (%) (*n* = 32)	Group 65–75 Years Old (%) (*n* = 41)	*p*
CEA				
≤4.7 µg/L	24 (36.4%)	15 (53.6%)	9 (23.7%)	0.01
>4.7 µg/L	42 (63.6%)	13 (46.4%)	29 (76.3%)	
NA	7	4	3	
LDH				
≤210 U/L	55 (78.6%)	25 (83.3%)	30 (75.0%)	0.4
>210 U/L	15 (21.4%)	5 (16.7%)	10 (25.0%)	
NA	3	2	1	
Albumin				
<35 g/L	27 (39.1%)	11 (38.0%)	16 (40.0%)	0.86
≥35 g/L	42 (60.9%)	18 (62.0%)	24 (60.0%)	
NA	4	3	1	
Platelets				
≤400 × 10^9^/L	63 (88.7%)	28 (90.3%)	35 (87.5%)	0.71
>400 × 10^9^/L	8 (11.3%)	3 (9.7%)	5 (12.5%)	
NA	2	1	1	
Glucose				
≤7 mmol/L	26 (50.0%)	13 (54.2%)	13 (46.4%)	0.58
>7 mmol/L	26 (50.0%)	11 (45.8%)	15 (53.6%)	
NA	21	8	13	
Creatinine				
≤130 µmol/L	61 (89.7%)	24 (80%)	37 (97.4%)	0.02
>130 µmol/L	7 (10.3%)	6 (20%)	1 (2.6%)	
NA	5	2	3	

**Table 3:** Cancer treatments in patients with metastatic colorectal cancer over 80 years old versus patients 65 to 75 years old.

Treatment	All (%)	Group > 80 yo (%)	Group 65–75 yo (%)	*p*
Previous Neoadjuvant Chemotherapy (Rectosigmoid/Rectal)				
Yes	11 (55.0%)	1 (16.7%)	10 (71.4%)	0.02
No	9 (45.0%)	5 (83.3%)	4 (28.6%)	
Previous Adjuvant Chemotherapy (Stage II)				
Yes	6 (60.0%)	1 (20.0%)	5 (100.0%)	0.01
No	4 (40.0%)	4 (80.0%)	0	
Previous Adjuvant Chemotherapy (Stage III)				
Yes	20 (80.0%)	12 (80.0%)	8 (80.0%)	1.00
No	5 (20.0%)	3 (20.0%)	2 (20.0%)	
Palliative Chemotherapy				
Yes	33 (45.2%)	11 (34.4%)	22 (53.7%)	0.10
No	40 (54.8%)	21 (65.6%)	19 (46.3%)	
Lines of Palliative Chemotherapy				
1	21 (63.6%)	6 (54.5%)	15 (68.2%)	0.96
2	6 (18.2%)	2 (18.2%)	4 (18.2%)	
3	4 (12.1%)	2 (18.2%)	2 (9.1%)	
4	2 (6.1%)	1 (9.1%)	1 (4.5%)	
Previous Neo-adjuvant/Adjuvant Radiation (Rectosigmoid/Rectal)				
Yes	13 (65.0%)	3 (50.0%)	10 (71.4%)	0.36
No	7 (35.0%)	3 (50.0%)	4 (28.6%)	
Palliative Radiation				
Yes	16 (22.0%)	8 (25.0%)	8 (19.5%)	0.57
No	57 (78.0%)	24 (75.0%)	33 (80.5%)	
Previous Curative Surgery				
Yes	45 (61.6%)	25 (78.1%)	20 (47.6%)	0.01
No	28 (38.4%)	7 (21.9%)	21 (52.4%)	

**Table 4:** Chemotherapy adverse effects in patients with metastatic colon cancer over 80 years old versus patients 65–75 years old.

Adverse Effect	All (%) (*n* = 33)	Group > 80 yo (%) (*n* = 11)	Group 65–75 yo (%) (*n* = 22)	*p*
Fatigue				
Yes	11 (33.3%)	4 (36.4%)	7 (31.8%)	1.00
No	22 (66.7%)	7 (63.6%)	15 (68.2%)	
Neutropenia (Grade 4)				
Yes	3 (9.1%)	0 (0.0)	3 (13.6%)	0.53
No	30 (90.9%)	11 (100.0%)	19 (86.4%)	
Peripheral Neuropathy				
Yes	8 (24.2%)	2 (18.2%)	6 (27.3%)	0.69
No	25 (75.8%)	9 (81.8%)	16 (72.7%)	
Hand-Foot Syndrome/Skin Changes				
Yes	9 (27.3%)	5 (45.5%)	4 (18.2%)	0.12
No	24 (72.7%)	6 (54.5%)	18 (81.8%)	
Bowel Changes (Diarrhea, Constipation)				
Yes	18 (54.5%)	7 (63.6%)	11 (50.0%)	0.71
No	15 (45.5%)	4 (36.4%)	11 (50.0%)	
Nausea/Vomiting				
Yes	13 (39.4%)	4 (36.4%)	9 (40.9%)	1.00
No	20 (60.6%)	7 (63.6%)	13 (59.1%)	
Mucositis				
Yes	3 (9.1%)	1 (9.1%)	2 (9.1%)	1.00
No	30 (90.9%)	10 (90.9%)	20 (90.9%)	
Other (Fatigue, SOB, Bleeding)				
Yes	11 (33.3%)	6 (54.5%)	5 (22.7%)	0.12
No	22 (66.7%)	5 (45.5%)	17 (77.3%)	

**Table 5:** Comorbidities in patients with metastatic colon cancer over 80 years old versus patients 65–75 years old.

Comorbidity	All (%) (*n* = 73)	Group > 80 yo (%) (*n* = 32)	Group 65–75 yo (%) (*n* = 41)	*p*
Obesity (*n* = 73)				
BMI > 30	22 (30.1%)	4 (12.5%)	18 (43.9%)	0.005
BMI ≤ 30	51 (69.9%)	28 (87.5%)	23 (56.1%)	
Diabetes (*n* = 73)				
Yes	26 (35.6%)	9 (28.1%)	17 (41.5%)	0.33
No	47 (64.4%)	23 (71.9%)	24 (58.5%)	
Hypertension (*n* = 73)				
Yes	57 (78.1%)	27 (84.4%)	30 (73.2%)	0.39
No	16 (21.9%)	5 (15.6%)	11 (26.8%)	
Cardiovascular/Neurovascular Disease (*n* = 73)				
Yes	32 (43.8%)	21 (65.6%)	11 (26.8%)	0.002
No	41 (56.2%)	11 (34.4%)	30 (73.2%)	
Dyslipidemia (*n* = 73)				
Yes	40 (54.8%)	24 (75.0%)	16 (39.0%)	0.004
No	33 (45.2%)	8 (25.0%)	25 (61.0%)	

## Data Availability

The original contributions presented in this study are included in the article. Further inquiries can be directed to the corresponding author(s).
